# Telemedicine Awareness, Knowledge, Attitude, and Skills of Health Care Workers in a Low-Resource Country During the COVID-19 Pandemic: Cross-sectional Study

**DOI:** 10.2196/20812

**Published:** 2021-02-25

**Authors:** Muhammed Elhadi, Ahmed Elhadi, Ahmad Bouhuwaish, Fatimah Bin Alshiteewi, Amna Elmabrouk, Ali Alsuyihili, Ayiman Alhashimi, Samer Khel, Alsafa Elgherwi, Ahmed Alsoufi, Ahmed Albakoush, Abdulmuez Abdulmalik

**Affiliations:** 1 Faculty of Medicine University of Tripoli Tripoli Libyan Arab Jamahiriya; 2 Faculty of Medicine Tobruk University Tobruk Libyan Arab Jamahiriya; 3 Faculty of Medicine Al-Jabal Al Gharbi University Gherian Libyan Arab Jamahiriya; 4 Faculty of Medicine Libyan International Medical University Benghazi Libyan Arab Jamahiriya

**Keywords:** attitude, awareness, coronavirus, COVID-19, knowledge, pandemic, skills, telemedicine

## Abstract

**Background:**

Since the onset of the COVID-19 pandemic, several health care programs intended to provide telemedicine services have been introduced in Libya. Many physicians have used these services to provide care and advice to their patients remotely.

**Objective:**

This study aimed to provide an overview of physicians’ awareness, knowledge, attitude, and skill in using telehealth services in Libya.

**Methods:**

In this cross-sectional study, we administered a web-based survey to health care workers in Libya in May 2020. The questionnaire collected information on physicians’ general demographic characteristics, ability to use a computer, and telemedicine awareness, knowledge, attitude, and skills.

**Results:**

Among 673 health care workers who responded to the survey, 377 (56%) and 248 (36.8%) reported high awareness and high computer skill scores, respectively, for telemedicine. Furthermore, 582 (86.5%) and 566 (82.6%) health care workers reported high knowledge and high attitude scores, respectively. We observed no significant differences in awareness, knowledge, attitude, and skill scores among physicians employed at public, private, or both types of hospitals. We observed significant differences in the mean awareness (*P*<.001), attitude (*P*=.001), and computer skill scores (*P*<.001) , where the score distribution of the groups based on the ability to use computers was not similar. Knowledge scores did not significantly differ among the three groups (*P*=.37). Respondents with professional computer skills had significantly higher awareness (*χ*^2^_3_=14.5; *P*<.001) and attitude (*χ*^2^_3_=13.5; *P*=.001) scores than those without professional computer skills. We observed significant differences in the mean computer skill scores of the groups (*χ*^2^_3_=199.6; *P*<.001).

**Conclusions:**

The consequences of the COVID-19 pandemic are expected to persist for a long time. Hence, policy programs such as telemedicine services, which aim to address the obstacles to medical treatment owing to physical distancing measures, will likely continue for a long time. Therefore, there is a need to train and support health care workers and initiate government programs that provide adequate and supportive health care services to patients in transitional countries.

## Introduction

SARS-CoV-2 was identified as the cause of severe viral pneumonia in Wuhan, Hubei Province, China, in December 2019. The World Health Organization declared COVID-19, the disease caused by this virus, a pandemic in March 2020 [1-4]. As of July 22, 2020, more than 15,000,000 COVID-19 cases and more than 617,000 COVID-19–related deaths have been recorded in 213 countries and territories [5].

Preventive measures have been implemented to reduce the potential exposure of individuals to the virus and to decrease the burden of COVID-19 transmission. These measures include hand washing, avoiding touching the face and eyes, cleaning and disinfecting surfaces and objects, wearing protective equipment including masks, and most importantly, social distancing by staying at home, avoiding close physical contact with others, and maintaining adequate interpersonal distance [6-8].

The COVID-19 pandemic has affected traditional in-person health care services. New strategies and approaches, including telemedicine and telehealth services, must be implemented to facilitate video consultations and the use of mobile apps, with the goals of delivering medical advice, diagnosis, and treatment to reduce the risk of infection [9,10]. Telemedicine and telehealth services are especially important for individuals without COVID-19 seeking medical treatment during the pandemic, especially those with chronic or acute illnesses. Several countries had begun increasing the use of telemedicine services even prior to the COVID-19 pandemic [11-16]. Telemedicine was effective during previous outbreaks including those of the Ebola virus, Zika virus, and the severe acute respiratory syndrome coronavirus [17,18].

Telemedicine is defined as the use of web-based resources and electronic information along with advanced digital network technology to promote long-distance professional health services, disseminate medical safety reports, deliver health-related education to the public, and carry out public health monitoring [19,20].

Since the onset of the COVID-19 pandemic, several health care service programs aiming to provide telemedicine services have been introduced in Libya. Even with their recent introduction, many physicians have used these services to provide care and advice to patients remotely. There is a need to assess the awareness, knowledge, attitude, and skills of physicians who use telemedicine services. The skilled use of telemedicine by physicians benefits patients, who can receive a wide range of health care services without the need to visit the clinic. Telemedicine services can reduce the risk of infection among populations by facilitating social distancing and decreasing the burden on the hospitals that provide care to individuals with COVID-19 [21-23].

This study aimed to provide an overview of physicians’ awareness, knowledge, attitude, and skills of telemedicine—a technology that has been recently introduced in transitional countries. Furthermore, we investigated the effects of COVID-19 on this technology to determine whether it is adequately applicable during the ongoing COVID-19 pandemic.

## Methods

### Study Design

In this cross-sectional study, we administered a web-based survey to health care workers in Libya in May 2020 via text messages and email. Responses were anonymous without any identifying data. This study focused on health care workers who were officially registered with Libyan hospitals during the COVID-19 pandemic and were involved in patient care. The first section of the survey collected baseline demographic information and characteristics of the health care workers, including age, gender, years of experience, employment status, department of work, and computer skills, and there were several general questions related to telemedicine experience. The second section contained a survey instrument that was previously developed by Zayapragassarazan et al [24], which contains four detailed subsections.

#### Awareness Subsection

This subsection consisted of 12 items on telemedicine, which were used to assess awareness. Each item was assessed with a 3-point scale with scores of 0-2 (0=don’t know, 1=heard of it, 2=know about it). The overall score could range from a minimum of 0 to a maximum of 24.

#### Knowledge Subsection

This subsection consisted of 11 items that assessed the physician’s level of telemedicine knowledge. Each item was assessed with a 2-point scale with scores of 0 and 1 (0=yes, 1=no). The overall score could range from a minimum of 0 to a maximum of 11.

#### Attitude Subsection

This subsection consisted of 11 items used to determine the attitude of the respondents toward telemedicine. Each item was assessed with a 5-point Likert scale with scores ranging 0-4 (0=strongly disagree, 1=disagree, 2=undecided, 3=agree, 4=strongly agree). The overall score could range from a minimum of 0 to a maximum of 44.

#### Computer Skills Subsection

This subsection consisted of 13 items used to determine the physician’s level of information technology and computer skills. Each item was assessed with a 4-point scale with scores of 0-3 (0=unskilled, 1=learner, 2=mediocre, 3=expert). The overall score could range from a minimum of 0 to a maximum of 39.

### Assessment of the Effect of COVID-19 on Telemedicine

The third section included several questions on the effects of the spread of COVID-19 on telemedicine, the role of telemedicine in reducing in-person consultations, the time spent on practicing telemedicine, the usability of telemedicine based on local internet settings, and the role of telemedicine in helping physicians avoid or decrease the risk of infection potentially resulting from internal conflicts in Libya.

Raw scores of each subsection of the awareness-knowledge-attitude-skill questionnaire were converted to percentages and compared with other group variables. We considered scores of ≤49% as low, 50%-70% as average, and ≥71% as high. The survey was conducted in accordance with the Checklist for Reporting Results of Internet E-Surveys [25].

### Statistical Analysis

Descriptive statistics related to the general demographic characteristics of the physicians were calculated. Frequency and percentage values were used to describe these variables. Mean (SD) values were used to describe continuous data. None of the major continuous outcomes, including survey scores, were normally distributed. Therefore, we analyzed them using the Mann-Whitney *U* test to determine whether there were significant differences between the two groups and the score of each section. We performed the Kruskal-Wallis *H* test to compare groups with ≥3 independent variables. The chi-square test was used to determine potential associations between the categorical groups. Statistical analysis was performed using SPSS software (version 25.0, IBM Corp). A *P* value of ≤.05 was considered significant.

### Ethical Consideration

The study procedures comply with the ethical standards of the relevant national and institutional committee on human experimentation, with the Helsinki Declaration of 1975, as revised in 2008. Ethical approval (approval# 109.3-2020) for this study was obtained from the Bioethics Committee at the Biotechnology Research Centre (Tripoli, Libya). The survey was conducted anonymously, and all participants provided written informed consent before participating in the study.

## Results

### Telemedicine Awareness, Knowledge, Attitude, and Skills of Health Care Workers

From among 800 health care workers who received the survey, 673 responded (response rate=84.1%). Among them, 299 (44.4%) were male and 374 (55.6%) were female. The participant cohort comprised 288 (42.8%) specialists or senior physicians and 385 (57.2%) physicians in training. Table 1 summarizes the baseline demographic characteristics of the study participants.

Most participants (n=631, 93.8%) had an average or high level of computer and internet skills. Most participants (n=525, 78%) had participated in training programs on telemedicine during the COVID-19 pandemic. Most participants (n=639, 94.9%) expressed their willingness to participate in such training courses.

The questionnaires assessed the physicians’ awareness, knowledge, attitude, and skills of telemedicine. The assessment scales had a high level of internal consistency (Cronbach *α*=.823 for awareness, Cronbach *α*=.735 for knowledge, Cronbach *α*=.910 for attitude, and Cronbach *α*=.950 for skills).

Interestingly, 399 (59.3%) participants perceived telemedicine as a helpful tool for elderly patients, and 486 (72.2%) speculated that telemedicine could be used by physicians to obtain patients’ electrocardiography and x-ray reports for medical consultation. Some participants (n=178, 26.4%) speculated that telemedicine technologies can facilitate direct consultations. Only 28 (4.2%) participants speculated that laboratory findings could be shared with patients through telemedicine technologies, and only 81 (12%) participants speculated that follow-up and treatment response evaluation could be performed using telemedicine technology.

Many participants (n=580, 86.2%) did not agree that patients should have access to information regarding their medical condition. However, 573 (85.1%) participants believed that telemedicine could help bridge the gap between primary and secondary care by fostering communication among physicians, consultants, and nurses. Interestingly, 254 (37.7%) participants had little or no experience using emails and sending files.

Data on physicians’ awareness, knowledge, attitude, and skills of telemedicine are summarized in Table 2. In total, 377 (56%) participants had high awareness scores, 582 (86.5%) had high knowledge scores, 556 (82.6%) had high attitude scores, and 248 (36.8%) had high scores for computer skills of telemedicine technology for all specialties. Overall, 132 (19.6%) participants were not familiar with telemedicine technology, and 28 (4.2%) had low telemedicine knowledge. In addition, 16 (2.4%) participants had low attitude scores.

**Table 1:** Baseline demographic characteristics of the study participants (N=673).

Variables		Total	Females (n=374)	Males (n=299)	*P* value
**Age range (years), n (%)**					.007^a^
	<30	109 (16.2)	48 (12.8)	61 (20.4)	
	30-40	442 (65.7)	247 (66.0)	195 (65.2)	
	>40	122 (18.1)	79 (21.1)	43 (14.4)	
Experience (years), mean (SD)		8.78 (8.09)	9.6 (8.54)	7.76 (7.38)	.001^a^
**Employment status, n (%)**					<.001^b^
	Public sector	335 (49.8)	150 (40.1)	185 (61.9)	
	Private sector	82 (12.2)	49 (13.1)	33 (11.0)	
	Both	256 (38.0)	175 (46.8)	81 (27.1)	
**Ability to use computers, n (%)**					.004^a^
	Beginner	42 (6.2)	15 (4.0)	27 (9.0)	
	Average	452 (67.2)	246 (65.8)	206 (68.9)	
	Professional	179 (26.6)	113 (30.2)	66 (22.1)	
**Received training for telemedicine system, n (%)**					.37
	Yes	525 (78.0)	287 (76.7)	238 (79.6)	
	No	148 (22.0)	87 (23.3)	61 (20.4)	
**Availability of a telemedicine unit in your department, n (%)**					.61
	Yes	525 (78.0)	289 (77.3)	236 (78.9)	
	No	148 (22.0)	85 (22.7)	63 (21.1)	
**Department, n (%)**					<.001^b^
	Internal medicine (including all subspecialties)	179 (26.6)	101 (27.0)	78 (26.1)	
	Pediatrics	74 (11.0)	33 (8.8)	41 (13.7)	
	Surgery (including all subspecialties)	160 (23.8)	131 (35.0)	29 (9.7)	
	Gynecology and obstetrics	72 (10.7)	3 (0.8)	69 (23.1)	
	Dermatology	36 (5.3)	11 (2.9)	25 (8.4)	
	Family medicine	64 (9.5)	38 (10.2)	26 (8.7)	
	Psychiatry	25 (3.7)	14 (3.7)	11 (3.7)	
	Other	63 (9.4)	43 (11.5)	20 (6.7)	

^a^*P*<.05.

^b^*P*<.001.

**Table 2:** Different levels of awareness, knowledge, attitude, and skills among health care workers in Libya (N=673).

Degree	Awareness	Knowledge	Attitude	Skills
Low (≤49%), n (%)	132 (19.6)	28 (4.2)	16 (2.4)	226 (33.6)
Average (50%-70%), n (%)	164 (24.4)	63 (9.4)	101 (15.0)	199 (29.6)
High (≥71%), n (%)	377 (56.0)	582 (86.5)	556 (82.6)	248 (36.8)

Table 3 compares awareness and knowledge scores, and Table 4 compares the attitude and skill scores among the study participants. The Mann-Whitney *U* test was performed to investigate significant differences in the awareness, knowledge, attitude, and computer skill scores between males and females. Females had significantly higher mean awareness (*P*=.02) and computer skill scores (*P*<.001) than males. However, we observed no significant differences in the mean knowledge (*P*=.55) and attitude (*P*=.99) scores between males and females.

**Table 3:** Differences in the awareness and knowledge scores of the study participants based on baseline demographic characteristics (N=673).

Variables		Total, n (%)	Awareness scores (range 0-100)			Knowledge scores (range 0-100)		
			Mean (SD)	*U*^a^ or *H*^b^ value	*P* value	Mean (SD)	*U* or *H* value	*P* value
**Gender**				49926.5^a^	.02^c^		54460.5^a^	.55
	Male	109 (16.2)	69.9 (24.8)			83.0 (16.4)		
	Female	442 (65.7)	66.5 (23.5)			83.5 (17.1)		
**Employment status**				2.79^b^	.25		2.16 ^b^	.34
	Public sector	335 (49.8)	68.0 (24.9)			83.1 (16.6)		
	Private sector	82 (12.2)	73.1 (21.2)			86.0 (13.9)		
	Both	256 (38.0)	67.5 (24.3)			82.6 (17.7)		
**Ability to use the computer**				41.97^b^	<.001^d^		1.98^b^	.37
	Beginner	42 (6.2)	49.6 (28.1)			77.3 (24.9)		
	Average	452 (67.2)	67.1 (23.8)			83.3 (16.2)		
	Professional	179 (26.6)	76.3 (21.4)			84.6 (15.5)		
**Department**				8.99^b^	.25		10.68^b^	.15
	Internal medicine	179 (26.6)	67.4 (25.9)			82.4 (17.3)		
	Pediatrics	74 (11.0)	65.6 (24.6)			83.7 (15.3)		
	Surgical specialties	160 (23.8)	66.9 (26.2)			80.8 (19.6)		
	Gynecology and obstetrics	72 (10.7)	73.4 (18.2)			85.6 (15.8)		
	Dermatology	36 (5.3)	70.4 (20.4)			85.6 (12.4)		
	Family medicine	64 (9.5)	73.5 (20.8)			86.4 (15.2)		
	Psychiatry	25 (3.7)	73.7 (24.5)			89.5 (8.2)		
	Other	63 (9.4)	64.3 (24.5)			82.3 (15.2)		

^a^Mann-Whitney *U* test for pairwise comparison of independent variables.

^b^Kruskal-Wallis *H* test for multiple-group comparison of independent variables.

^c^*P*<.05.

^d^*P*<.001.

**Table 4:** Differences in the attitude and computer skill scores of the study participants based on their baseline demographic characteristics (N=673).

Variables		Total, n (%)	Attitude scores (range 0-100)			Computer skill scores (range 0-100)		
			Mean (SD)	*U*^a^ or *H*^b^ value	*P* value	Mean (SD)	*U* or *H* value	*P* value
**Gender**				55878.50^a^	.99		43475.50^a^	<.001
	Male	109 (16.2)	79.8 (12.7)			56.4 (25.3)		
	Female	442 (65.7)	79.8 (14.3)			66.1 (22.3)		
**Employment status**				1.93^b^	.38		4.17^b^	.12
	Public sector	335 (49.8)	80.3 (13.5)			60.1 (24.8)		
	Private sector	82 (12.2)	80.7 (13.4)			66.1 (20.2)		
	Both	256 (38.0)	78.9 (13.7)			62.7 (24.3)		
**Ability to use the computer**				13.46^b^	.001^c^		199.62^b^	<.001^d^
	Beginner	42 (6.2)	72.6 (15.7)			37.9 (21.6)		
	Average	452 (67.2)	79.6 (13.3)			55.8 (20.5)		
	Professional	179 (26.6)	81.9 (13.3)			82.4 (19.3)		
**Department**				16.67^b^	.02^c^		22.87^b^	.002^c^
	Internal medicine	179 (26.6)	78.3 (13.9)			59.8 (25.8)		
	Pediatrics	74 (11.0)	79.6 (13.6)			56.5 (25.6)		
	Surgical specialties	160 (23.8)	78.9 (15.7)			67.1 (21.9)		
	Gynecology and obstetrics	72 (10.7)	83.1 (10.5)			56.5 (23.8)		
	Dermatology	36 (5.3)	80.4 (14.0)			55.3 (25.4)		
	Family medicine	64 (9.5)	82.8 (13.2)			63.6 (22.7)		
	Psychiatry	25 (3.7)	83.1 (8.9)			71.9 (24.7)		
	Other	63 (9.4)	78.4 (10.9)			64.1 (23.1)		

^a^Mann-Whitney *U* test for pairwise comparison of independent variables.

^b^Kruskal-Wallis *H* test for multiple-group comparison of independent variables.

^c^*P*<.05.

^d^*P*<.001.

We performed the Kruskal-Wallis *H* test to investigate differences in the awareness, knowledge, attitude, and skill scores of other categorical groups, including those based on employment status, ability to use computers, and departments of work. We observed no significant differences in the awareness (*P*=.25), knowledge (*P*=.34), attitude (*P*=.38), and computer skill (*P*=.12) scores among health care workers employed at public, private, or both types of hospitals. We observed significant differences in the mean awareness (*P*<.001), attitude (*P*=.001), and computer skill (*P*<.001) scores, where the score distribution based on the ability to use computers was not similar among these groups. We observed no significant differences in the knowledge scores of the three groups based on employment status (*P*=.34). Participants with professional computer skills had significantly higher awareness (*χ*^2^_3_=14.5; *P*<.001) and attitude (*χ*^2^_3_=13.5; *P*=.001) scores than those without professional computer skills. Furthermore, we observed significant differences in the mean computer skill scores among different groups (*χ*^2^_3_=199.6; *P*<.001). Overall, those who were more capable of using computers displayed significantly higher computer skill scores than those who were lesser capable of using computers.

We observed significant differences in attitude scores (*χ*^2^_8_=16.7; *P*=.02) and computer skills (*χ*^2^_8_=22.9; *P*=.002) among health care workers in different departments. In particular, those working in the departments of gynecology and obstetrics, family medicine, and psychiatry had significantly higher attitude scores (*P*=.02), while those in the department of psychiatry and those in surgical specialties had significantly higher computer skill scores (*P*=.002) than those of their counterparts in other departments. However, awareness (*P*=.25) and knowledge (*P*=.15) scores did not significantly differ among health care workers in different departments.

### Effects of COVID-19 on Telemedicine

Among the study participants, 638 (94.8%) thought that telemedicine technology could be used to limit the spread of COVID-19. Additionally, 630 (93.6%) participants thought that telemedicine could help reduce hospital visits to avoid COVID-19 transmission. However, only 283 (42.1%) participants thought that telemedicine could replace traditional medical visits during the COVID-19 pandemic. In addition, 616 (91.5%) participants thought that the telemedicine system could facilitate patient communication and cooperation with physicians during the COVID-19 pandemic. Furthermore, 622 (92.4%) participants thought that the use of telemedicine and remote health care systems saved a lot of time that was otherwise lost in hospitals and clinics and that these remote services could more rapidly provide medical advice. However, only 437 (64.9%) participants thought that internet services in Libya met the demand for uninterrupted telemedicine services. Moreover, 121 (18%) participants thought that telemedicine could help physicians avoid the issues of conflict and civil war in Libya and decrease the risk of infection arising from these internal conflicts. Interestingly, 575 (85.4%) participants thought that telemedicine could potentially prevent physicians who worked in unsafe environments from sensing insecurity during the civil war. Additionally, 606 (90%) participants thought that telemedicine could help physicians avoid the risk of contracting and transmitting COVID-19. However, only 385 (57.2%) participants thought that the telemedicine service technologies would help improve the financial status of physicians.

## Discussion

### Principal Findings

Telemedicine has been introduced recently in transitional countries, including Libya, to provide remote health care services, especially to patients with chronic diseases. In this study, 377 (56%), 582 (86.5%), and 566 (82.6%) physicians responding to our survey had high awareness, knowledge, and attitude levels for telemedicine, respectively; however, only 248 (36.8%) participants had adequate or high computer skills.

The COVID-19 pandemic has placed greater emphasis on telemedicine technology as a means of providing adequate care to patients without increasing the risk of SARS-CoV-2 transmission to patients during in-hospital clinic visits. Additionally, telemedicine benefits health care workers because it decreases the risk of infection among them, reduces stress in the hospital, provides an adequate tool for treatment and follow-up evaluation of their patients, and provides a suitable approach for providing mental health services.

This study provides an overview of physicians’ awareness, knowledge, attitude, and skill levels of telemedicine in a transitional country that is afflicted with a civil war, a financial crisis, and a devastating health care system during the COVID-19 pandemic [26-28].

Only 179 (26.6%) participants had professional computer skills, while 452 (67.2%) and 42 (6.2%) participants had average and beginner computer skill levels, respectively. Thus, overall, the computer and information technology skills of the physicians were inadequate. Such skills are crucial for the productive use of telemedicine services. Therefore, we recommend software and computer skill training programs for physicians who are newly introduced to telemedicine technology in transitional countries. Nonetheless, the approach may be difficult even with these skills. Among all our participants, 236 (35.1%) indicated that the internet services in Libya were not sufficient to meet patient needs through telemedicine technology owing to electricity and internet interruptions in Libya after the civil war [29]. This remains yet another challenge for telemedicine service providers, requiring immediate attention from internet providers and telephone companies [30]. Many patients have complained about the unavailability of these services owing to their high demand during the COVID-19 pandemic.

Most study participants (n=556, 82.6%) had higher attitude scores. This is important because attitude represents how telemedicine was perceived by health care workers [31]. The attitude of health care workers is an important factor in understanding and accepting telemedicine technologies. For such acceptance, the program developer needs to train health care workers and make the telemedicine programs usable for them [32].

Knowledge and attitude are important for the acceptance of telemedicine by health care workers. In this study, 582 (86.5%) participants had high knowledge scores; this value is higher than that reported previously in Germany (where approximately 63% of individuals had some telemedicine knowledge) [33]. Another study conducted in Lagos [34] reported that only 60.9% of participants had telemedicine knowledge. Furthermore, another study conducted in Pakistan [35] reported an average level of telemedicine knowledge among health care workers. Further initiatives and commitments are required to expand the use of telemedicine and increase its efficiency, especially among health care professionals in a postconflict setting. There is a need for greater understanding of telemedicine among health care practitioners, particularly those who are new telemedicine users. Hence, it is important to address the financial and physical access–related obstacles to telemedicine [36].

There are several constraints in the implementation of telemedicine in transitional countries, especially in emergency settings, notwithstanding the implementation of strategies to reduce the risk of exposure to the virus during the COVID-19 pandemic. Certain technical issues and the need to train health care workers to provide adequate telemedicine services need to be addressed. The COVID-19 pandemic has put a strain on hospital health care workers and has posed challenges to politicians, managers, and practitioners with regard to the limits of the broader health care infrastructure and assumptions that have restricted its potential for swift and innovative reforms [30,37,38].

### Limitations

Our study was limited to Libya; hence, our findings may not be generalizable to the populations of other countries. Furthermore, the cross-sectional design of our study does not allow for drawing concrete conclusions establishing causation between variables. In addition, our study does not provide details regarding the types of telemedicine technologies and how they can be implemented, along with patients' attitude toward telemedicine. Further studies on telemedicine are required to provide qualitative measures of health care services to patients, to assess the access to health care services in other transitional countries, and to determine whether these telemedicine adaptations benefit patients, especially those with chronic illnesses and psychiatric morbidities [39-41]. Additionally, there is a need to determine the cost benefit of these services and whether modifications are required in these services.

### Conclusions

The consequences of the COVID-19 pandemic are expected to be long lasting. Hence, policy programs, such as telemedicine services, which aim to address obstacles to medical treatment owing to physical distancing measures, are also required to be provided over a long term [42]. Therefore, there is a need to train and support health care workers and initiate government programs that provide adequate and supportive health care services to patients in transitional countries and to respond to issues regarding the access to these services, including internet access and advertisements, and social programs that help patients understand how to use these services.
